# Supplementary material for: Predicting visual function by interpreting a neuronal wiring diagram
Source: Nature. 2024 Oct 2;634(8032):113–23. doi: 10.1038/s41586-024-07953-5 (PMC11446822; doi:10.1038/s41586-024-07953-5)
Supplement: Supplementary file 6 — CRF and ERF predictions for individual TmY4 and TmY9 cells. Analogous to Supplementary Data 3, but for TmY target types. Shown are the top four monosynaptic pathways, the strongest pathway passing through each of the top ten intermediary types (ranking from Extended Data Fig. 7), and the trisynaptic pathway Tm1–TmY–Dm3–TmY (see the section entitled Prediction of spatial normalization). [file 41586_2024_7953_MOESM6_ESM.zip › DataS4/TmY9q/720575940631323719.pdf]

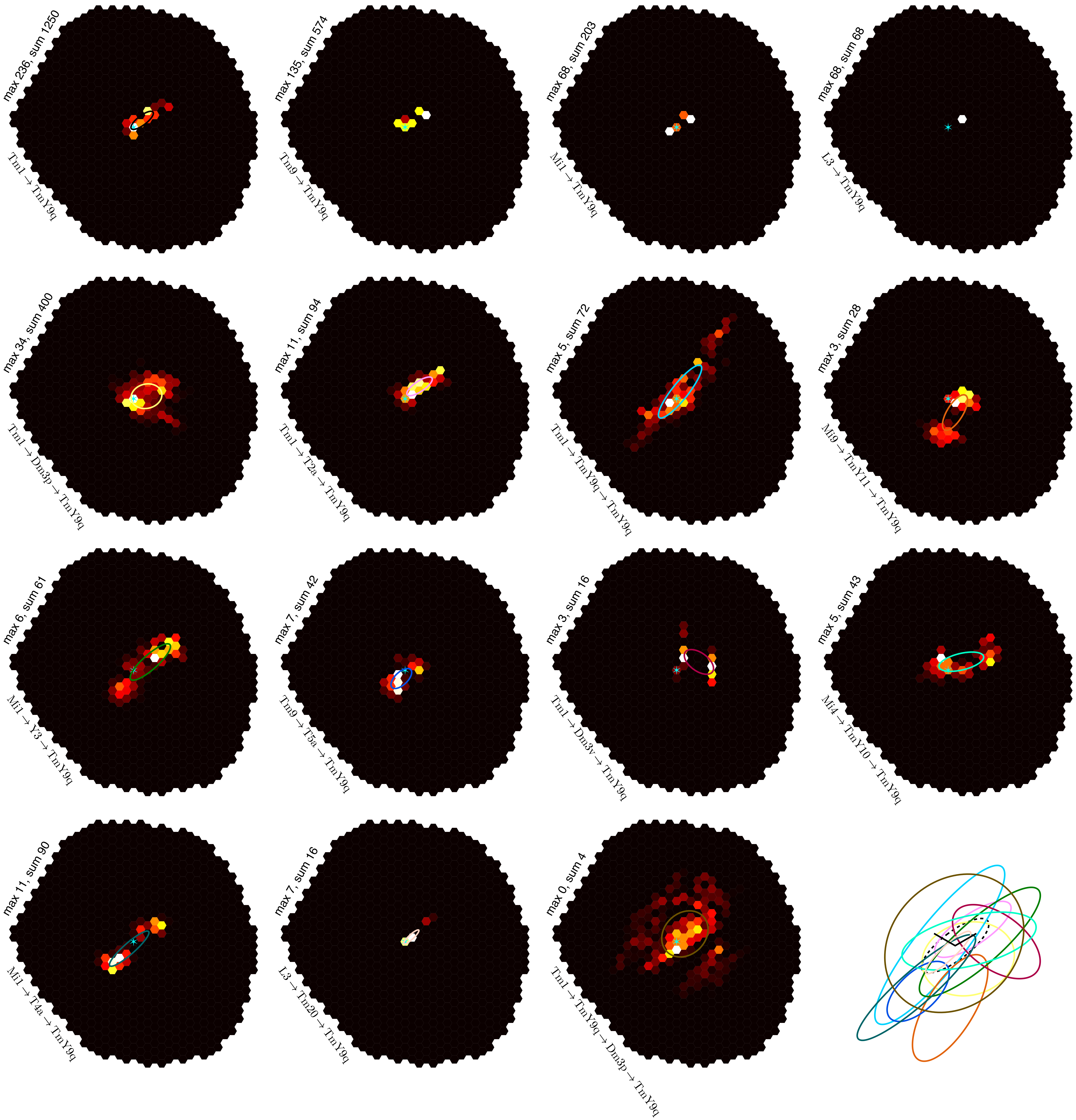
max 236, sum 1250
Tm1 → TmY9q
max 135, sum 574
Tm9 → TmY9q
max 68, sum 203
Mi1 → TmY9q
max 68, sum 68
L3 → TmY9q
max 34, sum 400
Tm1 → Dm3p → TmY9q
max 11, sum 94
Tm1 → T2a → TmY9q
max 5, sum 72
Tm1 → TmY9q → TmY9q
max 3, sum 28
Mi9 → TmY11 → TmY9q
max 6, sum 61
Mi1 → Y3 → TmY9q
max 7, sum 42
Tm9 → T5a → TmY9q
max 3, sum 16
Tm1 → Dm3v → TmY9q
max 5, sum 43
Mi4 → TmY10 → TmY9q
max 11, sum 90
Mi1 → T4a → TmY9q
max 7, sum 16
L3 → Tm20 → TmY9q
max 0, sum 4
Tm1 → TmY9q → Dm3p → TmY9q
